# Key Tenets of Operational Success in International Animal Welfare Initiatives

**DOI:** 10.3390/ani8060092

**Published:** 2018-06-09

**Authors:** Michelle Sinclair, Clive Julian Christie Phillips

**Affiliations:** Centre for Animal Welfare and Ethics, School of Veterinary Science, University of Queensland, Gatton, Queensland 4343, Australia; c.phillips@uq.edu.au

**Keywords:** animal welfare, strategy, international, culture, stakeholders, success

## Abstract

**Simple Summary:**

With increasing frequency, animal welfare organisations are looking to operate in multiple countries. Apart from lessons learned by trial and error and personal experiences informally shared, little formal knowledge is available to support the challenging task of achieving success for the animal welfare movement beyond borders. Through gaining interview insights from leaders of some of the world’s biggest international animal welfare organisations, this study was able to conduct an analysis to find reoccurring themes and important concepts relating to both successful and unsuccessful initiatives, and investigate the reasons behind their success level. The prominent findings are presented and aim to be of use when looking to develop international animal welfare strategy.

**Abstract:**

Animal welfare is an increasingly global initiative, which makes the intricate business of operating across borders of particular relevance to the movement. There is, however, a distinct absence of literature dedicated to investigating operational strategies that are more likely to result in the success of international animal welfare initiatives. In addition to this, opportunities exist to investigate the human aspects of animal welfare, parallel to the growing field of animal-based science. This study aimed to begin addressing these gaps by conducting semi structured interviews with 15 leaders of some of the largest international animal welfare charities. Leaders were asked to describe their experiences of successful and unsuccessful initiatives within the animal welfare movement. Thematic analysis was then conducted to identify recurring concepts and extrapolate potentially applicable information. Engaging stakeholders and communities in locally-led and culturally respectful ways were discussed, as was the importance of knowledge, moderation, flexibility, and mutual benefits. The dangers of attacking personal and cultural identity are also highlighted and discussed. Key quotes and examples are presented, supplemented with mind maps as a tool to more readily apply the findings of the study in strategy development.

## 1. Introduction

Following the path of commercial enterprise, not-for-profit social initiatives are increasingly seeing the globe through a lens of borderless possibility. The growth of technology and issue awareness through social media, together with an increasingly affluent world, means opportunities exist that have not been seen before. Residents of countries with higher GDPs are increasingly seeking to share wealth and fortune for a better world [1], in causes such as reducing poverty, providing health care and education opportunities where they did not exist before, and addressing racial inequality, gender empowerment, environmental protection, and animal welfare. However, running international initiatives poses many risks; the risk that the issue will not progress, that resources will be lost, that the initiative will not be wholly successful, that relationships will be damaged, and perhaps worst of all, the risk that the initiative will evoke negative reactions and will set the issue backwards, rather than progressing it. While operating in a global landscape brings many potential rewards, it does not come without major challenges for not-for-profits; primarily attributable to specific social progression issues being viewed differently in different areas of the globe, with differing associated importance levels. ‘Animal Welfare’ is one of those issues, only with the added challenge of being a social cause not centered around humans. This can be a particular challenge when considering projects run in lesser developed countries, by charities based in higher developed countries. Numerous psychological models suggest that people care about activities according to their personal level of need [2,3,4]. Maslow proposed a hierarchy of needs that required the lower levels to be satisfied (such as shelter, food, and water) before higher levels such as self actualisation and purpose. Although this conventional model has endured to become mainstream within psychology, Maslow later updated his pyramid to place ‘self-transcendence’ at the top; a motivation to look beyond the self, and to act in service of others and something bigger [5]. More recently, others have updated the pyramid, such as the ‘updated hierarchy of fundamental human motives’ [2], but in each case, base psychological needs outplay other needs, and in some instances, the ability to focus on social progression and advocate for others is only a motivational aspiration when all other needs are met. For some, the ability to focus on charitable causes is a luxury.

Although these psychological models were developed in Western countries, it would be reasonable to consider that it may be more difficult to elicit a care for the wellbeing of animals for a mother struggling to feed her crying baby, or a government body preoccupied with safe sanitation of waterways. In total, 11% of humans are currently classified as ‘hungry’, with that number growing [6]. Clean water is a problem for 2.6 billion people (https://water.org/our-impact/water-crisis/), residents of many countries are dealing with governing bodies that are corrupt [7], and climate change is a crisis increasingly affecting all people [8]. For these reasons, animal welfare often takes an international back seat where policy, funding, and attention are considered. However, if considering non-human animal lives from a utilitarian framework, and with associated value, the scale of suffering globally is highly significant. The planet now holds around three chickens to every human, with 9.2 animals per person being farmed for human consumption each year [9,10], often in ways considered by science to cause great suffering. While the numbers of domestic animals living within human societies continue to increase, it is now also argued that all species on earth, including those that are living naturally in the wild, are now impacted by human society [11]. In addition to the scope for suffering amongst non-human animals, people are increasingly concerned about animal welfare. A recent study showed that ‘animal protection’ ranked highly when rated by importance, even against other social issues such as poverty, racial equality, and the death penalty [12]. The animal welfare movement is growing, and has claimed major wins all over the world; the abolition of battery cages, sow stalls, veal crates, and animal cosmetic testing in some countries factor amongst the biggest. The ethical argument to actively address animal welfare issues is strong; however, when dealing with far less resources than human-based initiatives, and the added challenges to advocating for another species with whom many people struggle to relate, animal welfare charities need to be clever. The need to run international animal welfare initiatives carefully and with a knowledgeable strategy becomes of paramount importance. ‘For profit’ causes have been investing time and money into researching how best to do this within their sectors, developing best practices and methods that will work to see a goal to fruition. ‘Market research’, for example, is conducted to fully understand what products will work, and the ways in which to present them for increased uptake. However, a distinct lack of literature and evidence exists to support the work of charities. This is particularly the case regarding animal welfare charities where the added challenges and dimensions of advocating for another species are perhaps not addressed in human welfare centric literature.

Animal welfare initiatives are anecdotally too often trial and error and most charities have notoriously felt they have needed to operate in a competitive silo, rarely learning from the mistakes and successes of others. This does not mean that integral knowledge does not exist within the movement. Over decades, long term animal welfare leaders and bodies have inevitably become better through this process of trial and error. This strategic information, however, is rarely available to the animal welfare movement in general. Exceptions to this generalisation do, however, exist. Arranged networking forums such as international conferences, chance meetings between leaders, and the publication of a few industry-focused books by long term leaders operational in the field, aim to capacity-build the movement. In general, however, the literature focused on succeeding in an international environment is both extremely limited and lacks comparative analysis. This may represent an opportunity for improvement through a more coordinated approach within the animal welfare movement. Analysis of what works for different leaders may increase the chance of developing generic strategies which can be successfully employed by an increased number of other leaders.

To this purpose, this study aims to begin to understand the key tenets of successful international animal welfare initiatives, as shared by international animal welfare leaders across organisations and regions. It aims to build themes based on the frequency of appearance when describing both successful and unsuccessful programs, and to further investigate some of the details of those themes. The overall purpose of this study is to provide information to animal welfare leaders seeking to operate successful initiatives across borders, and to aid in the creation of best practices for international animal welfare initiatives.

## 2. Method

Ethical approval for the study was provided by the University of Queensland Human Ethics Committee (approval number 2017000628). Relevant organisations were considered to be the major international animal welfare charities on the basis of three aspects of their activities: international, large scale, and a high level of brand recognition. In total, the leaders of 13 major international animal welfare organisations were approached in October 2017 for interviews in October or September of the same year. Leaders from 10 organisations accepted, one declined, and two did not reply to invitation. The reason for declining the invitation by one organization was a perceived lack of knowledge to comment on the subject. Participating organisations were Animals Asia, Humane Society International, Compassion in World Farming, International Animal Rescue, International Fund for Animal Welfare, World Animal Protection, Society for the Protection against Cruelty to Animals UK, Vets Beyond Borders, The Donkey Sanctuary, and People for the Protection of Animals.

The leaders of the organisations were chosen by the researcher and organisations based on their role within the organization, which required them to be a Chief Executive Officer (CEO), operations managers, or high-level co-ordinators working in an international capacity. These leaders were approached via email and asked to take part in a 30 min semi-structured interview via Skype to talk about their experiences regarding successful and unsuccessful projects in animal welfare. Prior to the interview, the leaders were given an information sheet to review, outlining the confidentiality of the interview, the voluntary nature of the interview, their right to withdraw at any time, and details in regards to the topic and interview logistics such as the time requirement and platform. Upon request, all leaders also gave verbal permission at the conclusion of the interview to use the name of their organisation as participating within the study. Fifteen leaders accepted this invitation to participate and a time was booked for interview.

In the interview, leaders were asked:

- To describe the most successful international animal welfare projects they have been involved in.
- To describe the least successful international animal welfare projects they have been involved in.
- What made those projects successful/unsuccessful.
- How long they have been in animal welfare.
- What drew them to animal welfare.
- What makes a good international animal welfare leader.

Responses from all interviewees to those questions marked in italics are presented in this paper, with the remaining to be presented in a later paper to allow for complete discussion and logical division of data. All interviews were recorded with a voice recorder after verbal permission from each leader at the onset of the interview, and complete transcripts were subsequently prepared verbatim. This study was approached in a familiar way to sociological research across fields (including that of other social progress initiatives such as environmental conservation), with the purpose of identifying and understanding evidence-based approaches as solutions for advancing the movement [13,14,15].

### Data Analysis

Thematic analysis was conducted using NVivo (QSR International, http://www.qsrinternational.com/products_nvivo-mac.aspx, Melbourne, Australia), and through manual inspection of source data, by the same researcher that conducted the interviews (MS). Best practice sociological research methods for approaching and analyzing qualitative data within thematic analysis and creating grounded theory were engaged with this dataset [16]. This included the use of NVivo to ‘manage’ data which can then be manually coded into key themes [17] using ‘text frequency’ functions within the program to identify the most commonly utilized words. ‘Word search’ functions were then used to display the text before and after the word to assist in further identifying the context in which the frequent words were being used. Coding themes were ultimately selected for deeper anlaysis after being identified using these text frequency and word search functions in NVivo, in addition to manual familiarisation with the data. Words were chosen for analysis based on the amount of times they appeared overall (through NVivo); however, joining words (such as ‘and’) were excluded, along with words that drew no relevance or usefulness to the node/theme or study (selected manually). By extracting the main themes that emerged from the analysis, including key pieces of connective information and direct quotations, mind maps were created to visualize the themes and their relationship with each other. Data within each ‘node’ [17] (identified reoccurring theme) was then further analysed for more detail, including frequency of theme and importance.

## 3. Results

**Figure 1:**
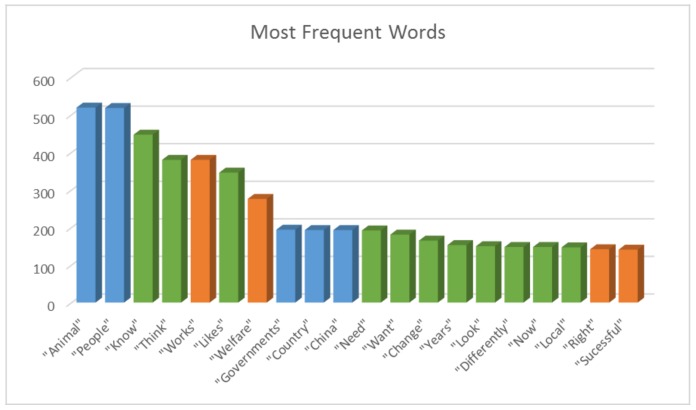
Bar graph visually presenting the 20 most frequent words used by respondents when describing successful international initiatives. Note: Data points are colour coded depending on the context in which they are mostly found; nouns (who) are indicated by blue, instructive actions (how) by green, and outcomes (what) by orange.

Figure 1 and Table 1 represent the most frequent words used by study respondents when describing successful initiatives. 

**Figure 2:**
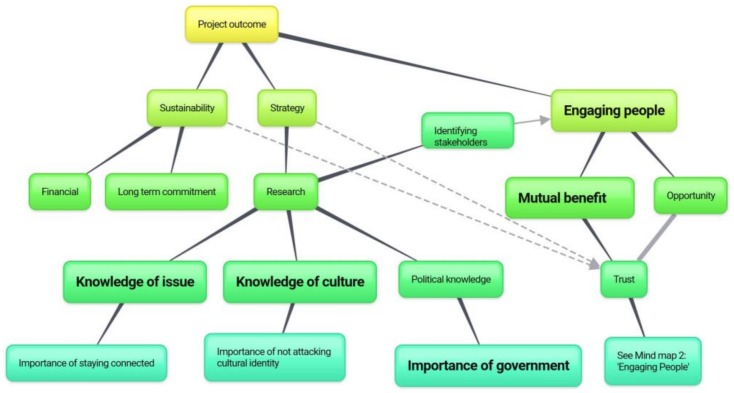
Tenets of successful international animal welfare initiatives. Note: Bold text indicates a frequent theme (appearing in more than 70% of interviews). A solid line indicates a strong relationship between themes, with dotted lines indicating a present relationship of significance of lower frequency (therefore strength).

**Figure 3:**
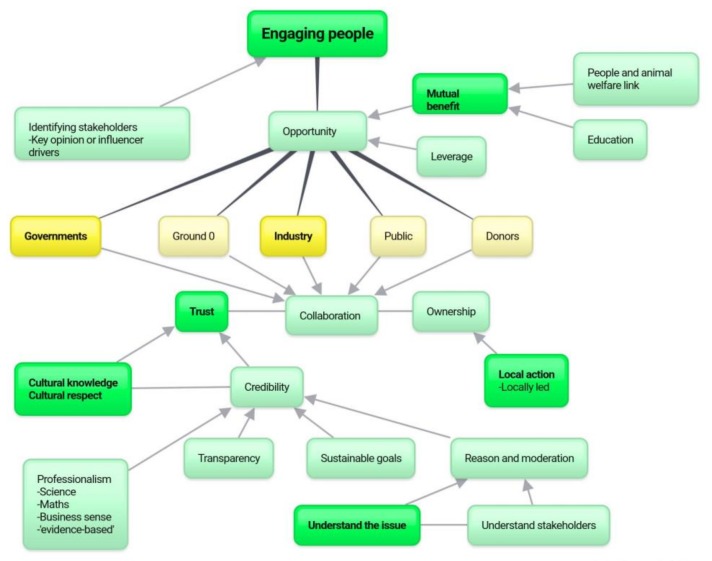
Engaging people. Note: Bold text indicates a frequent theme (appearing in more than 50% of interviews). ‘Ground 0’ refers to stakeholders that are in a position to make direct choices (positive or negative) regarding the welfare of animals (i.e., farm workers, slaughtermen etc.).

As reflected in Table 2, sustainability, strategy, and engaging people were the base themes to success. The themes flowing from these indicate routes or notions of importance in support of the key themes (Figure 2). Research and the knowledge gained from this research are central to many successful themes, with ‘engaging people’ to find a mutual benefit (and opportunities to engage), leading to trust-building activities (bolstered by sustainable strategy) underpinning this model. Given the complexity and importance of ‘engaging people’ within this study, a further mind map was created to focus specifically on this theme and the themes that tie into it (Figure 3).

### 3.1. Sustainability

The key theme of sustainability appeared 69 times and in 11 out of 15 interviews (Table 2). Sustainability here mostly referred to the importance of achieving long term sustainability, and second to that, allocating sufficient finances to ensure that longevity is possible. This also included having plans that enabled the allocation of necessary resources. In relation to projects in Asia, one leader stated that “no one should be coming into this continent if they’re not prepared for a long, long slog”. Others commented that their major success was mainly due to ‘persistence’; another attributed their success to ‘trust built with governments because they have shown that they are in for the long haul’, contrasting this with an NGO that has ‘great plans, but only for 2 years’: “It’s not a quick fix, you cannot just walk into a country, have a couple of meetings and that’s it. It takes time, just like with everything, it takes time to develop that relationship and trust”. Three leaders cited 10–20 years as a time frame in which they have seen significant change. One leader stated that “the impact we are able to drive for animals today is essentially harvesting fruit from the trees that were planted 20–30 years ago; if we want systemic change tomorrow, we need to be planting the acorns, the thought seeds today, to get that big change”.

### 3.2. Strategy

Although the word ‘strategy’ did not frequently appear in the interviews, many other themes emerged that can be classified as strategy building and/or important to building an effective strategy, particularly in the earlier stages of creating initiatives and campaigns. The importance of having a holistic strategy was raised by eight of the leaders while discussing reasons behind successful initiatives (Table 2), in comments such as “we realised our end goal was for (the) African elephant to be protected, but then we have to stop every (supply and demand) link on the chain”, and “the continuity of our work with government, with traditional medicine, with all the stakeholders actually… our consistent involvement, our sensitivity for the politics, for the culture, for the plight of the people involved in the industry as well”. Some leaders also referred to the lack of a holistic strategy in the context of failures: “(in) India, when I first started I tried to engage directly with the egg production industry, and we even found some people from some companies, brought them to the US for a tour of how cage free is done so they could learn—which is a training program we still run—but I just offered it with nothing else in place right… there was no pressure at that point from the government, there was no public policy pressure for them to change, the market wasn’t really asking for it”. 

Interviewees commonly cited the importance of having knowledge when creating a strategy (14/15 leaders); however, the specific knowledge that was required varied in nature. Firstly, knowledge required them to fully understand the issue they were looking at addressing (11/15 leaders), including the relevant political landscape and a knowledge of the key stakeholders (4/15 leaders). The dangers of not identifying the right stakeholders were emphasized: “sometimes when you go into a country it is hard to identify who is the right stakeholder to engage… (describes an involved initiative that aimed to demonstrate improve farm animal practices)... it was a waste of money... as ultimately they had no influence of policy, and no influence over the actual farmers”; and “As a movement, we need to become better at identifying who the stakeholders are”. It was also commonly perceived that there was a specific cultural and/or country knowledge (12/15 leaders, Table 2), which was strongly tied to engaging people and is explored in more detail in that theme. Lastly, when analysing the text associated with strategy, the word ‘focus’ was mentioned by over half the leaders, appearing 13 times (Table 1). The idea of focusing on a key mandate was present more frequently, with comments such as “I’ve learnt you should try to do a few things really well, instead of doing everything badly”. This suggests that excessive diversification can be a reason for a project being unsuccessful. 

### 3.3. Engaging People

All international leaders raised the importance of engaging people, including stakeholders, in initiatives (see Table 2), and the specific word (and word extensions) ‘engage’ was used 72 times (Table 1). It was the most prevalent theme (276 references, Table 2), and the theme attributed most to successes where engagement was achieved, and to failures when it was missed or not achieved. Although only eight of those leaders used the specific word ‘engagement’, all produced data that was understood during interview and analysis stages to fit the definition of engagement as attributed to the act of being engaged, and defined as “the process of encouraging people to be interested in the work of an organization” [18]. 

#### 3.3.1 Cultural Knowledge and Respect

In the context of engaging people, key themes emerged as integral to this process. The most prevalent was ‘cultural understanding and respect’ (12/15 leaders, 43 references, Table 2), with the word ‘culture’ (and extensions such as ‘cultural’) used 84 times (Table 1). One leader explained the primary reason behind their successes as follows: “this team take root in the country and understand the society because we know China has his own characteristic”. Others expressed frustration over initiatives that appeared to have failed due to a lack of understanding of the culture and country, or in the case of a wanton application of a campaign element that had seen success in another country with a very different culture. Commenting specifically on the application of initiatives from Western-based cultures into Eastern-based cultures, one said, “there are just political, economic, cultural, and social boundaries or environmental restrictions that means animal welfare projects in Asian countries may not meet the expectation or standard, whatever that standard is, (the standard) that western people think it should be; it needs to be mutual understanding, but also, let go”; and another stated, “If you train people you have to trust people to take the concept and interpret it in their way, (a way) that’s suitable for that culture and that political environment”. The need to understand culture was not limited to West and East, rather, there is a need to fully understand the culture across any borders: “It’s very easy to assume from here that Ireland is a bit like the UK isn’t it, but actually no, it’s very different culturally in terms of animal welfare ethics”. 

An important element of this theme was how language, and specifically the concept of animal welfare, translates in that culture. It was reported that the Chinese word for ‘animal’ is a ‘moving object’, making discussing the welfare of ‘moving objects’ decidedly more difficult. In addition, another leader stated, “when you’re translating ‘animal welfare’ it doesn’t translate (easily) into Vietnamese; it translates very badly”. Attitudes that concerned some leaders included ‘what about my welfare, my welfare (needs) haven’t been met’. One leader stated, “how can we talk about animal welfare (when) people see welfare as a luxury, that you give, and most people don’t have—or felt that they don’t have”. In response to this attitude, one leader began by changing the language: “so we changed IFAW’s name, when I translate (the new name) back it is ‘International Fund for Love and Care of Animals’, so, by doing that, we basically put the responsibility to people to provide the very basic needs for animals, so, welfare changed in peoples’ minds from a luxury thing to something that’s basic”.

#### 3.3.2 Attacking Cultural Identify and Perceived Rights

In contrast to this, in describing failed initiatives, a similar but new theme emerged. Although rarely directly acknowledged as such in the data, we consider that it should be labelled ‘attacking perceived rights and cultural identity’ of stakeholders (9/15 leaders, 19 references, Table 2). Examples were failed initiatives to stop cultural festivals and activities involving animals (bull-taming and cock fighting festivals were specifically mentioned). Interviewees also reported the described stakeholders‘ responses to the variety of external pressures that were applied, and indicated that their defensiveness solidified cultural identity. One example, the level of dog meat consumption in Korea, achieved the direct opposite to the goal the campaign had intended. Further comments emphasized the issue of personal rights: “it comes down to the fact that it’s very much a campaign of attacking someone’s personal beliefs and someone’s personal rights… people are always going to be more concerned about how they’re being attacked and their belief is being attacked over welfare of animals”. Other comments included “it doesn’t benefit from international condemnation”, “people are very protective of their culture”, “I think we somehow presume that because we find something horrible, that everyone else should find it horrible, and not understanding that there is a fairly strong cultural identity”, and “I think a lot of people who are sitting on the fence would then say ‘you don’t come into a country and tell us what to do, now we‘re going to be on the side of the people who are carrying out this practice, even if we don’t agree with it”, thereby consolidating this theme. One Asian leader drew a passionate parallel by stating, “boycotting doesn’t work, pointing that finger from the outside doesn’t work; who likes being told what to do? Who would like a bunch of Koreans, Vietnamese and Chinese coming into the UK and saying you shouldn’t be badger baiting… It’s a very dangerous circumstance to be that imperialistic and arrogant to tell another country what to do”. One leader stated that they were careful what they publish on one specific country; China, as “every time China has, you know, an animal cruelty story, the Western media jump on it and use it as a China bashing tool”, the consistent sentiment being that this was counterproductive, as are the repercussions: “you come in with a Western dictating attitude you will be shut out by the government, and it is very easy to shut you out”. The examples of negative outcomes when attacking identity were again not limited to West and East. One leader spoke about Canada, in relation to the seal hunt: 

“It seems like the most horrible thing, like, (a) baby seal clubbed to death. As in, who would possibly think that it is defendable anyhow? However that particular fishing tribe in Canada might see that their identity is under attack, and now they’re being portrayed as a villain, and they kind of want to attack back by saying what we’re doing is right, and there again there is no scope for collaboration because the person does not want to speak at all. I’m not saying you collaborate to the point that you lose your identity as an animal welfare organisation, I’m just saying that just being a little more thoughtful before we launch a campaign against a community, especially if this is done by a certain set of people, one needs to be really, really careful”.

One solution proposed was to find a positive way forward, by first understanding the reasons for the practice, and how people feel about it: “it has been their culturally accepted practice over the years, you can’t go there and shout at them what they’re doing is wrong, you’ve got to first understand why they’re doing it, and once you understand it you need to be articulate and be very very sympathetic on how you present your point”. Responses suggest that this must be underpinned by a strong cultural knowledge and respect, and that we should look to alternative languages such as mathematics (budget gains) and science (credibility via professionalism) instead of emotion and cultural attacks, particularly when approaching governments and industry. Then, initiatives have a chance to be led “from the inside out”.

#### 3.3.3. Importance of Being Locally Led

Tied with the themes of ‘cultural knowledge and respect’, and as a counter theme to ‘attacking cultural identity’, is the notion of having initiatives ‘from the inside out’: “you have to start at a local level and work up”. ‘Local’ (and it’s extension ‘locally’) appeared 147 times (Table 1), with the theme being raised by 11/15 leaders (Table 2). One leader cited the need to represent issues in a ‘locally inspired way’ as the key motivator behind setting up the charity they lead: “I just felt that these issues needed to be represented from a local perspective rather than an international perspective; myself and local Chinese founders decided we wanted to move ahead and address the issues from the people who really understood living and working in Asia, rather than the outside in. We wanted it inside out”. Another leader judged the success of a program by the amount of local groups that get involved in the cause or campaign, and another described “the successful ones are actually (the ones that) allow the local animal lovers, or local people, to take ownership of the project and not just not create the term animal welfare, but not create so much boundaries… give them the basic understand then let them use their creativity”. 

In one example, a leader described a successful primate rescue initiative that was set up in a way that it was not only entirely locally run, but also engaged the entire community into the centre as primate babysitters, crop growers, security, and cleaning; people that were originally hunting the primates. Supporting programs to be locally led through capacity building appeared a few times through the interviews as successful measures to enable local action: “they help all the local people and show them as they’re working to up-skill them, and that’s been a huge success for us”. Tied to being locally led and supporting from ‘behind the scenes’ is capacity building to help to build local ownership. 

One leader remarked on the importance of not being too protective about ownership and taking credit when working beyond borders. This may seem counterintuitive to the usual charitable model, which often relies on the ready acceptance of credit to justify asking for donor support. However, it may allow for greater success of the animal welfare initiatives on the ground, across the movement: “We just sat back and said ‘well done, oh what a good idea you’ve had’… it did help embed things more clearly with them and promote ownership… the other thing that helps enormously is not to be too precious about ownership and credit”.

Lastly, in addition to engaging and collaborating with local people, some leaders attributed a big part of their successful campaigns to ensuring their staff are all local: “you know, this whole idea of stakeholder involvement, you really can’t get anything done in a country like Indonesia, without being totally immersed in it, and that means employing people”. Another, when describing reasons for allocating success to an initiative, stated that “the leadership of that office from top to bottom, they’re staffed by people in that country”; “You have to have local people engaged and buying in to what you’re doing and then be the spokespeople for it”; and “I don’t hire anyone except Filipino for Philippines, Bangladeshis for Bangladesh, Nepalis in Nepal, I have, you know, the organisation believes capacity building and empowering local communities is the only way you will make your programs sustainable”.

#### 3.3.4 Mutual Benefit

The second most common identified sub-theme under the topic of engaging people in improving animal welfare, is the importance and benefit of finding mutual benefits (11/15 leaders, 35 mentions, Table 2). While some explicitly stated that a program will fail if ‘mutual ground’ or ‘mutual benefits’ are not identified, others referred to it indirectly. Leaders made comments such as “you can’t go in asking for something, you have to go in giving something, and that’s the only way you’re going to get (what you want)”; “I think again that comes down to trying to find a benefit for those people in… the campaign”; “Real sustainable change comes from changing hearts and minds and… identifying a common interest/ solution is by far the most effective way to do this”; and “Come up with an incentive… don’t persuade them to do something they don’t want to do, offer them something”.

The potential mutual benefits raised included commercial opportunity (farming, eco-tourism), job opportunity, improved public image or brand, financial reward, improved productivity, national pride, and even entertainment. One leader discussed engaging a village community by providing a peddle-powered cinema that would play movies, interspersed with conservation messages that would feed into their bigger picture of animal rescue goals: “It’s a novel way of getting their interest… the parents love their children on the bikes peddling like mad, the kids love it, they all queue up”. In another program that effectively ended the practice of dancing bears in India, career-changing education and training was given in direct exchange for relinquishing bears that would dance for income: “These people were depending on this bear for a livelihood, so how on earth could we just take that bear away and remove that livelihood?”… “in the end they weren’t even getting enough money to feed themselves, let alone their bear or their family, so I think we hit it at the right time”.

Another leader described a successful campaign that found unique mutual benefit through morals, with consumers of animal entertainment; offering enlightenment around the suffering of the animals when involved in the industry. The success of the campaign was attributed to finding a mutual ground of love for the animals that the consumers were being entertained by. 

One leader outlined novel thinking in regards to finding mutual benefits and collaborating with other charitable causes, outside animal welfare, to broaden the bigger picture impact of the animal welfare movements. Discussing an animal rescue project being conducted in developing areas of Africa, they stated, “by partnering with people who may be concerned about water, they may be concerned about livelihoods, they may be concerned about gender issues, all of these things would help us find partners that are trying to achieve one or the other or usually multiply the sustainable development goals”.

Benefit for governments to become involved in improving animal welfare was raised by a few leaders, particularly through budget-saving initiatives and improved human health and disease control. A leader described a successful approach to government: “all I asked was ‘show me your budget that you’ve spent for the last five years on culling’… they showed me and I said ‘what if for a quarter of this I’m able to show you better results in the next five years and I guarantee there will be no more dog bites than you had in the past five years because I know it works that way”.

#### 3.3.5. Importance of Working with Governments and Industry

Nearly all of the leaders (13/15, Table 2) mentioned the importance of working with governments, either as a key component of a successful initiative, or as the primary reason an initiative failed or had great troubles. The word government (and it’s extensions ‘governance’ and ‘governments’ etc.) appeared within the top 10 most used words (194, Table 1): “I think the major thing is… you need to be working with local government and local authorities”…“Because the government has so many powers in this country… they could launch policy… they could launch new laws to make people to (sic) do this, do that”.

A few leaders stated that government engagement itself was a strong indicator of the success of an initiative, with one stating that it was ‘the end game’: “To have the government standing up and inviting (us) to be their partners in bringing it (bear bile farming) to an end in the next five years”. Another leader attributed one of their most successful initiatives, the rabies vaccination and spay of 70% (also referred to as community saturation) of animals across an entire country, to their partnership and long term collaboration with the royal government of the country. Others indicated a failure to work with the government as the key reason behind the overall failure of an initiative…“I guess they failed because they were not able to adapt to a political environment, or because they couldn’t come to an agreement or understanding of each other”. Partnering with the government to offer workshops was flagged as a sustainable option, where it fit the initiatives: “If you encourage the government and give them advice and give them successful experience and also other examples, they could learn from it, and they could do a lot of good things”.

In addition to working with the government, many of the leaders also described success in which they worked with industry, not against it: “Our consistent involvement, our sensitivity for the politics, for the culture, for the plight of the people involved in the industry as well”…“I think, just recognising you’re not working with an enemy here”. Another leader stated that “our other most successful program has been our corporate engagement work, where over the last 10 years we have worked with more than 800 companies in European Union, USA, South America and Australia, that work has been focused on driving commitment to game changing alterations to company product lines and supply chains; more than 1.1 billion farm animals are now set to benefit as a result of those interventions”. More than one leader described identifying commercial champions, or ‘insider champions’, for animal welfare improvement initiatives, who lead a corporate trend, as others fear ‘being left behind’: “Leadership was now with the companies and that this was a trend and it was gathering strength all the time”. One leader suggested that in some cases, where governments are now failing to act, that industry themselves can take leadership, with great success: “It’s the corporate agenda where we’ve been getting more change”.

During analysis of the text, collaborative approaches rather than taking confrontational attitudes (Table 2) appeared as a theme across the board, but particularly when discussing working with industry (8/15 leaders, 22 citations, Table 2: “We’ve never ever shut the door with talking to adversaries”…“It’s not like these poultry industry leaders wake up in the morning and say ‘let’s be cruel’ right? It is something that has become natural because of what the business is, and I feel we (must) start making some business sense to them”. One leader summed up the sentiments of collaboration by stating, “even though people are performing acts one sees as egregiously cruel and wrong, ethically wrong, you cannot classify them as the enemy, and working to understand how we can reverse their practices with the help of the government so that everyone wins, you know, bears, stakeholders in the industry, and the government too, so no one loses face in this continent where face is everything”.

#### 3.3.6. Trust and the Language of Reason and Moderation

Another frequently reoccurring theme within the interviews was that of trust (8/15 leaders, 20 word appearances, Table 2): “I think in terms of stakeholders, trust is just the most important issue… (and) I think that looks different depending on, you know, what you’re doing”. Another stated, “just trying to work with people to tell them you’re an ally not an enemy would be really important, no matter at what level you’re operating”. In describing their most successful program, a community horse care program, one leader reflected on where they began: “in the beginning I think they were largely mistrusting of the help… so, just sustained efforts (were made) to ensure we were trusted by the community, ensure that people know us there”.

To the purpose of extracting practical information that may be useful to this study and its application, this theme was intuitively followed and the data was then analysed for methods by which trust was being built by successful leaders. Again, themes emerged. These themes centered around building trust with the two most frequently mentioned stakeholders; industry and governments. As these are arguably the most powerful stakeholders, the consensus on engaging them appeared to be around creating credibility through professionalism, which included meeting them on their level and speaking in languages they understand: “We were the first international agency that agreed to put in our money as well as theirs, so I think there was a lot of trust built and that somehow I would put trust between each of us as partners as the number one factor, and the fact is of course that we never said no to anything that was expected and they reciprocated when times were tough for us, they reciprocated with certain requests that we had”. 

In the context of building trust, demonstrating capacity for long term presence through sustainable plans was also raised (as discussed in ‘strategy’). 

The importance of carefully-selected language—that of reason and moderation, appears emphatically in the interviews from a few key leaders when describing successful trust building relationships with governments. A scientific and business approach, based on knowledge, was advocated instead of a purely emotional approach: “When you’re talking to governments, they don’t expect or won’t deal with people who are ‘puppy-huggers’ you know?”. Another stated, “how we presented ourselves was (as a) scientific, professional unit, who knew what we had to offer with very proper protocols of monitoring and carrying out impact assessment... there were meetings being held, reports submitted, graphs being prepared, surveys being carried out; so a lot of activities which were unconventional to what an animal welfare organisation usually does”. Another leader stated, “I think what’s worked with us is we have used the global language of mathematics; we don’t speak in English or any local language, we let statistics, we let science and we let published information of the impact we’ve had to do the talking, and that’s why we’re successful”. In discussing reasons for a particular success, another leader stated, “I think… a key factor (was) being able to present ourselves as a capable, professional unit which actually knew what we were talking about, and we could display and prove what we were offering was actually going to be true”. 

Anecdotally, emotionally fueled lobby is often used by communities and the general public when reporting concerns to governments. With governments also in a position to be concerned with the difficulties that change may present for industry in greater recognition of the animals’ needs, opportunity exists for animal welfare professionals to support governments in moving forward by presenting scientific measurement and alternative but useful information and solutions. Emotional responses may be useful in engaging the general public to be interested in a cause, and indeed, generating donor generosity; however, caution is recommended when using emotion as a lone language even in this context of general public engagement. Emotional responses can be measured scientifically, e.g., to video footage of livestock slaughter [19] and their degree of representativeness investigated. Humans naturally empathasize with animals with poor welfare, and this response can be measured and informative [20]. In this respect, opportunity exists to present measured emotion as scientific evidence to audiences in which this language is more successful, such as governments and industry, as suggested in this study. However, it is also important that we have scientific validation around public concern about animal welfare, because the public may not be sufficiently knowledgeable about animal management [21].

To this, another leader in this study remarked on finding the language as the critical point in any stakeholder trust relationship: “So, getting a message that worked for them via an approach through people they trusted, we managed to gain access, but that was the critical point. The rest is relatively straight forward”. 

Speaking on the use of reason and logic as a language with governments against dog culling, one leader stated, “I give the example of how countries got rid of polio; (it is) not by killing the children who were born, but by vaccinating the children who are born… it’s the same way you can get rid of rabies in your country, by vaccinating the dogs and not killing the dogs, otherwise the logic is the same. Every animal that is born will have rabies, every child that is born will have polio… you kill the disease, you don’t kill the vector. Logic sets in well”.

Two leaders made special mention of ensuring that animal welfare initiatives have enough professional expertise to meet stakeholders at their level and to speak their language: “Companies are hiring the best scientific, economic business minds; how are we going to rise up to them if we don’t do the same thing?”

In addition to reason as a language of trust, moderation; the ability to be flexible, compromise, and reciprocate, was raised a few times in the interviews. This was particularly in the context of unsuccessful initiatives, due to an inability to be moderate. After probing some leaders to reflect on why campaigns to end dog meat have been so unsuccessful in some Asian countries (after they had raised the issue), these reasons centered on the inflexibility of the campaign, the defensiveness it generates in cultural identity (discussed in ‘attacking cultural identity), and the unsteady philosophical ground it finds itself on when compared to eating other species domestically and internationally (i.e.,—the response of “but we eat pigs”) [22]. Instead of taking the more extreme ground of ending dog meat consumption, perhaps this campaign requires a more moderate approach, to begin to see difference: “Let’s improve standards, you know, let’s look towards, creating better conditions for animals that we raise to eat”. Rather than taking extremist angles that are harder to support, and ignite defensiveness to identity, it is suggested that a “focus, and evidence-based common sense approach to achieving the goal” is preferred.

One leader, who had made a transition from a more aggressive ‘animal rights’ organisation, to a more moderate ‘animal welfare’ organisation, discussed the increased success that the change had made in relation to the actual impact on animals. Although vegan, this leader stated, “in relation to food sovereignty, I think the reason we have been successful has been… positioning ourselves in the centre, not leaning either to the right or to the left of the food sector”. The leader also discussed how more ‘moderate’ groups are able to collaborate with important stakeholders to bring about change, where more ‘extreme’ groups may not, and how the position of adversarial groups is often demonstrated in the war-like language; ‘’When groups put out press releases saying it’s a ‘victory’, ‘we did this’… the moment someone announces a victory there is a loser right? And nobody likes to be loser right? So they rise up to bring your victory down. So to me it seems like having press releases that don’t use the word ‘victory’ are a key, and collaborating and having conversation with adversaries so that it’s a win for them, a win for us and a win for the animals”.

The primary themes that appeared within the context of ‘engaging people’ in animal welfare initiatives are presented in Figure 3, commencing with identifying stakeholders (a product of research as indicated in Figure 2), and finding opportunity through identifying mutual benefits, which itself relies on understanding and investigating how the animal welfare issue is related to people and their welfare. ‘Education’ around the intrinsic importance of the issue (i.e., ethical positions etc.), separate to how it is linked to people, is offered as an alternative to finding the link; however, education of this nature is a long term endeavour. Having identified the stakeholders and the opportunities through mutual benefit, initiatives should be collaborative, which relies on trust and local ownership. Trust can be built on individual and organisational credibility, which includes a deep cultural knowledge, and integrally, respect. In the case of attacking cultural identity (outlined in the above section, and placed in Figure 2), trust is dissolved and success is unlikely. 

**Figure 4:**
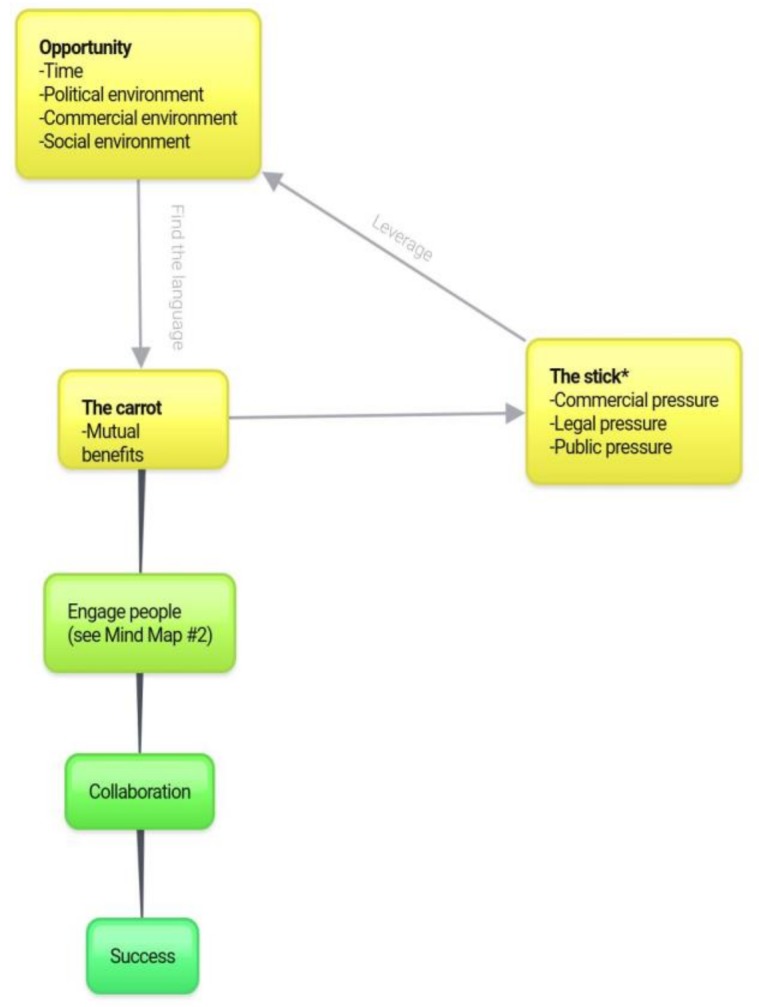
The case of the carrot vs the stick. * Asterisks highlights ‘the stick’ as an outlying strategy presented in the data, but one that was suggested to have use in specific circumstances, presented below.

In regards to engaging people, although the data suggest an approach almost entirely positively focused on ‘the carrot’ strategy, some argument for ‘the stick’ was present (Figure 4). Deeper analysis of the data was conducted in an attempt to understand in what context this approach may be useful. As demonstrated in Figure 4, the data tends to suggest that the positive approach is almost entirely the best practice; however, where mutual benefits do not exist or cannot be identified, pressure can be placed (through a variety of routes) to offer leverage and thus create opportunity. This opportunity can then be used to utilise mutual benefits and continue in a positively focused collaboration, rather than adversarial initiatives. Other ‘carrots’ may exist, such as better meat quality from animals with higher welfare; however, these are not individually listed as they are considered as a mutual benefit.

## 4. Discussion 

### 4.1. Applications

The predominant themes and recurring discussion items centered around ‘people’ words (‘people’ being the second most used word throughout, Table 1), and in specific relation to engagement, notions such as knowledge and respect. That is, knowledge of the issue, and even more so, knowledge of the people the issue concerns. In this context of international animal welfare project management, cultural knowledge and respect were of particular importance. When deconstructed, people drive the change that not-for-profit initiatives wish to see, but within the context of animal welfare, the focus is overwhelmingly placed on the animals: “It’s the people that we need to convince, the animals have no role to play in this”. There has often been a lack of people that have this perspective in the animal welfare movement, and a potentially misguided passion for animal welfare that without the ‘people’ focus, runs the risk of lacking the ability to bring about change—or worse—being detrimental to the movement. In many cases, sentiments and themes found in this study support conventional wisdom; however, not-for-profit organisations (focused here on animal welfare) are also susceptible to underusing the cross-disciplinary knowledge that is available in the sciences, business, marketing, psychology, international business management, and cross cultural research. This may be for a number of reasons, including a mistrust of science, the cost of accessing and appraising the information, and a lack of experience and skills in this area [23]. However, this common pitfall also represents an opportunity to improve the animal welfare movement. By utilising and applying knowledge gained by other fields, and by learning lessons from the successes and failures of long established leaders in the field, the efficacy of international animal welfare initiatives can only improve [24].

This study aimed to illuminate the latter, by consulting leaders on strategy, identifying themes behind success and failure, and considering the major beliefs behind these. Using this information, and the way the themes are tied together in the data, logical mind maps have been created and presented throughout the results to assist the digestion of this information and increase the application of findings. These mind maps may aid in constructing an improved international animal welfare strategy, as a checklist to ensure the comprehensiveness of a created strategy, and may aid in the diagnosis of an unsuccessful strategy.

### 4.2. Limitations and Further Research

While this study aims to commence research on best practice approaches to international animal welfare initiatives, further research is needed. This study was limited to the leaders of the major international animal welfare charities, which meant a sample size of just 15. While the data offered by these leaders is rich in experience and detail, further research could focus in more detail on country specific information. Opportunities also exist to analyse existing projects objectively for the presence of these themes, and to add themes and operational strategies where appropriate. Opportunities also exist to apply this information in case studies, and to measure their success to validate and elaborate on these themes. As noted by one of the interviewees in this study, “*I think that another need in the region is to just have more research on just how to engage, and also on the cultural aspects of animal welfare*”.

## 5. Conclusions

Within this study, leaders of the world’s largest international animal welfare not-for profits were interviewed with respect to strategic initiatives they considered to be successes and failures. It then investigated the key tenants behind successful and unsuccessful initiatives for themes and solutions, and developed approaches through mind maps for application to international animal welfare initiatives in the future. There is a tendency in animal welfare charities to fail to utilise research and methods tried and tested by other industries to improve operational efficiency. The animal welfare movement could benefit from an increasingly strategic focus, and a focus on people, as the stakeholders able to bring about change. How best to engage people, particularly in an international context, was investigated in this study. It is envisaged that the information within this study will prove useful in the creation of international animal welfare initiatives with improved efficacy, and provide a base for, or contribution to, the generation of best practices to be built through further research. Learning from successes and failures across the animal welfare movement can only aid in collectively furthering the movement; through the focus on people, to the benefit of animals. 

## Figures and Tables

**Table 1:** Frequency of words associated with successful initiatives.

Word	Count	Similar Words Included *
Animal	519	animal, animals, animals’
People	518	people, peoples
Know	447	know, knowing, knows
Think	380	think, thinking
Works	380	work, worked, working, works
Likes	346	like, liked, likely, likes
Welfare	276	welfare, welfare’s
Governments	194	governance, government, governments
Country	193	countries, country
China	193	china, china’s
Need	192	need, needed, needing, needs
Want	181	want, wanted, wanting, wants
Change	165	change, changed, changes, changing
Years	153	year, years
Look	150	look, looked, looking, looks
Differently	148	difference, differences, different, differently
Now	148	Now
Local	147	local, locally
Right	142	right, rights
Successful	141	success, successes, successful, successfully, successive
Programs	141	program, programmed, programs
Kinds	135	kind, kindness, kinds
Help	134	help, helped, helpful, helping, helps
Seeing	129	see, seeing, sees
Time	129	time, timely, times, timing
Understand	128	understand, understanding, understands
Campaign	127	campaign, campaigners, campaigning, campaigns
Make	127	make, makes, making
Talk	113	talk, talked, talking, talks
Trying	112	tried, tries, try, trying
Saying	108	saying
Issue	106	issue, issues
Company	96	companies, company
Community	95	communities, community
Problem	94	problem, problems
Good	90	good, goodness
Markets	87	market, markets
Groups	86	group, groups
Internationally	86	internal, international, internationally
Cultural	84	cultural, culturally, culture, cultures
Able	83	able
Giving	80	give, gives, giving
Industry	80	industries, industry
Important	77	importance, important
Impact	76	impact, impacted, impacts
Find	75	find, finding
Projects	75	project, projects
Terms	74	term, terms
Organisation	72	organisation, organisations, organise, organised, organising
Engage	72	engage, engaged, engagement, engages, engaging
Humans	71	human, humane, humans
Policy	70	policies, policy
Sustained	69	sustain, sustainability, sustainable, sustainably, sustained
Involvement	66	involve, involved, involvement, involving
Support	65	support, supported, supporters, supports
Approach	63	approach, approached, approaches, approaching
Development	62	develop, developed, developing, development

Note: While further variations of words, and words with a similar meaning, exist, they were not presented in this table as they did not appear in the data with significance.

**Table 2:** Frequency of nodes identified to themes and subthemes as a proportion of total participants (*n* = 15) and number of times the theme was mentioned, according to the attributed hierarchy.

Key Theme	Frequency of Theme, Number of Respondents out of 15	Frequency of References of Theme	Sub Theme	Frequency of Sub Theme, Number of Respondents out of 15	Frequency of References to Sub Theme
Engaging people	15	276	Cultural knowledge	12	43
			Importance of local action	11	22
			Mutual benefit	11	35
			Reason and moderation	7	10
			Trust	8	20
			Working with industry	8	22
			Livelihoods—people and animal welfare link	7	16
			Attacking ‘rights’ or identity	9	19
			Understanding the ‘perpetrators’	6	10
			Carrot over the stick	4	5
			Credibility through professionalism	5	9
			Education	7	11
			Collaboration with other charities	5	7
			Social media	4	7
			Staying connected by being on the ground	3	5
Sustainability	11	20	Financial sustainability	2	3
			Long term commitment	11	16
Strategy	6	9	Knowledge	14	85
			Importance of government	13	40
			Focus	9	43
			Importance of holistic strategy	6	9
			Identifying stakeholders	4	6
			Global standards	3	3

Note: sources: number of interviewees out of 15 that mention this theme or idea. Reference frequency refers to the amount of times in the dataset that this theme or idea was mentioned. Key themes correlate with the coding function of parent nodes, and sub themes correlate with child nodes in NVivo.
